# SCUD - Smart Culinary Utility Device: Leveraging edge AI for battery optimization and operation cycles

**DOI:** 10.1016/j.ohx.2026.e00780

**Published:** 2026-05-12

**Authors:** Sesidhar DVSR, Chandrashekhar Badachi, Chandrashekar Nagawaram, Panduranga Chary Kondoju, Pavana Salunkhe, Sahil Kumar Chaurasia

**Affiliations:** a ECE Department, MVSR Engineering College, Nadergul, Telangana, Bharat, 501510, India; b EEE Department, Ramaiah Institute of Technology, VTU, Belagavi, Bharat, 590018, India; c ABB India, Hyderabad, Bharat, 500081, India; d Ford Motor Company, Dearborn, Michigan, 48124, United States; e MED, KLS Gogte Institute of Technology, VTU, Belagavi, Karnataka, Bharat, 590008, India; f ETE Department, Ramaiah Institute of Technology, VTU, Belagavi, Bharat, 590018, India

**Keywords:** Data driven model, Optimization, Li-ion battery, Edge computing, State of charge, Training and testing

## Abstract

Current day society pursues a lifestyle that is simpler, more efficient, reliable, faster, and progressively automated. Culinary Utility Device available in the market exhibit drawbacks including substantial investment costs, increased labor requirements, and excessive time consumption. Adding intelligence to the these devices may make once life happy. Embedded devices with limited resources are now poised to leverage machine learning techniques thanks to the convergence of Edge Computing and the Internet of Things (IoT). Traditional machine learning often demands significant computational resources for predictive tasks. TinyML, which focuses on Embedded Machine Learning, aims to transition a considerable portion of users from high-end devices to low-end gadgets. This paradigm strives to ensure the accuracy of learning models while enabling training and deployment on micro edge devices with resource constraints. It also seeks to optimize processing capabilities and enhance system resilience. This article provides an intuitive overview of Data-Driven State of Charge (SoC) estimation for Li-Ion Batteries. It begins by specification table followed by the hardware in context introducing the background of SoC estimation, followed by discussions on Hardware and signal , links to the design files and BOM in specific. The article delves into essential aspects related to Build and Operational instructions. In conclusion, the article addresses critical challenges and outlines a future road map.

## Graphical abstract

**Figure:**
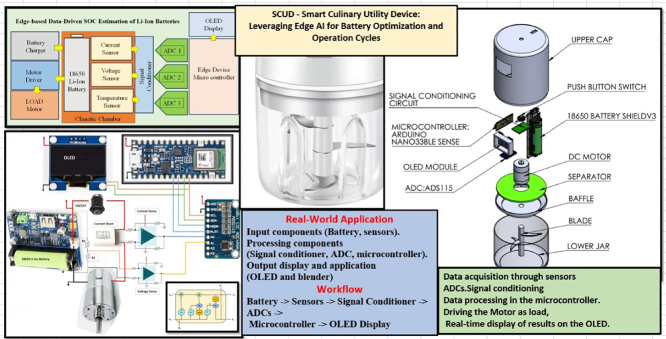

**Table:** 

Hardware name	SCUD - Smart Culinary Utility Device: Leveraging Edge AI for Battery Optimization and Operation Cycles

Subject area	Engineering and material science
Hardware type	Electrical engineering and computer science
Open source license	Creative commons attribution 4.0
Cost of hardware	₹1908.50 (INR)
Source file repository	https://osf.io/sq8pj/overview?view_only=ba723b95f7164bc7bca65879d7f6570b

## 1 Hardware in context

In recent years, home automation systems have been increasingly popular because they raise living standards and make daily chores more enjoyable, safe, and effective [1]. This study primarily focuses on a smart culinary utility device, frequently utilized in meal preparation. An Arduino Nano 33 BLE is utilized in this design, which can save a ML model along with the application code on its flash memory, and is powered by an 18 650 Li-Ion battery. All the other components are connected to the Edge device through connecting wires. This device can be used by manually hitting the button on the top lid. An OLED display shows the battery parameters, If the user wants to know the residual capacity of the energy source before it can be recharged again [2]. This design offers an easy-to-use and comprehends interface. The uses of lithium-ion (Li-Ion) batteries have been important in designing the rechargeable wireless applications, critical in ensuring both reliability and safety. In such batteries, competitiveness lies in the attractive promise of holding high storage capacity for energy, long life, efficiency, and environmental friendliness [3]. The key objective is to get reliable monitoring of batteries to ensure there is no abuse. It is crucial for battery management system to establish and maintain a connexion between the battery pack of the SCUD system and its overall performance. One important measure that characterizes the state of a battery is SoC, which shows the quantity of remaining power before the necessity of a recharge. The State of Charge (SoC) of Lithium battery cells is often represented using an equivalent circuit, which has thorough lookup tables for each circuit component. The battery’s performance at various operating points is determined by collecting experimental data using pulse discharge curves and charge curve measurements, these heavily rely on discharging processes and training characteristics such as Temperature, Voltage, and Current [4]. Techniques based on a data-driven approach focus on the input–output relationship and provide flexibility, nonlinear mapping, and adaptability. The data-driven SoC Estimation process consists of three main stages: Data Collection, Model Training, and SoC Estimation. Among some prominent techniques in this class are ANN, SVR, SVM, and FL [5], [6], [7], [8], [9]. The precision of these systems relies on the precise battery model and its parameters. Inconsistency of noise levels and the battery model’s structure lead to performance degradation.

## 2 Hardware description

**Fig. 1:**
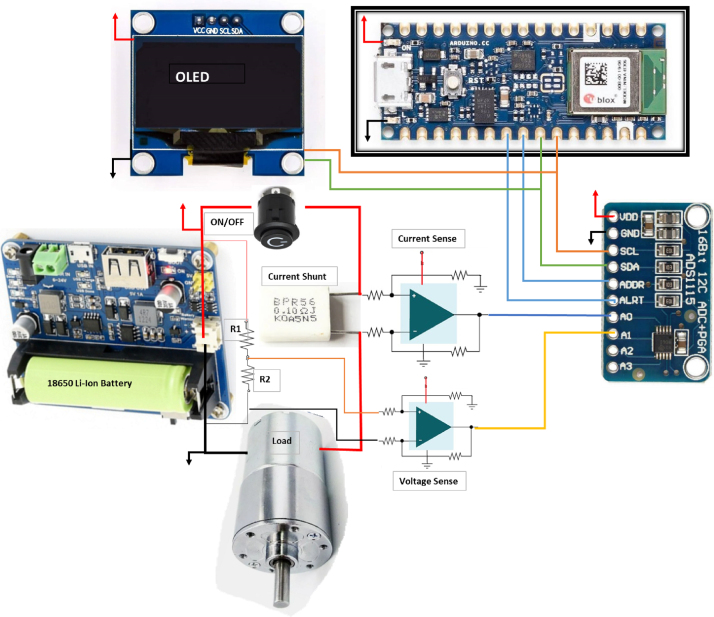
The battery management system.

State of Charge (SoC) estimation primarily involves the acquisition of critical data from a battery pack. This data encompasses various parameters, including voltage, current drain, cell temperature, battery charging and discharging rates, and power dynamics with respect to specific time stamps during its operational cycle. These parameters serve as the foundation for informed decision-making within smart battery management systems. The role of such systems is multifaceted. Firstly, they ensure the balanced operation of individual cells within the battery pack, especially in scenarios where charging and discharging profiles may vary significantly. This balancing prevents uneven wear and tear on cells, optimizing battery life. Secondly, smart battery management systems play a crucial role in averting thermal runaway situations. By monitoring and controlling temperature fluctuations, they safe-guard cells from overheating, which can lead to critical damage or even catastrophic failure. Furthermore, these systems are equipped to detect and manage faults in the battery’s operation promptly, ensuring safe and reliable performance. At the heart of this hardware system is the Smart Battery Monitor, an AI- powered Edge Platform. This monitor diligently tracks all pertinent battery parameters and offers valuable insights into SoC (State of Charge). Additionally, it has the capability to transmit data from the edge to cloud-based platforms if required when necessary while also maintaining local data storage for reference and analysis. Fig. 1 provides a comprehensive functional block diagram illustrating the Edge-based SoC Estimation process outlined above. In practical terms, SoC estimation is a critical component of battery management, ensuring that a battery’s performance aligns with its intended operational requirements. Factors such as operating voltage, current, charge/discharge history, and, when relevant, temperature conditions all contribute to determining the state of charge (SoC) of a battery. The State Of Charge (SoC) of batteries is a crucial use-case that indicates their remaining power before requiring a recharge. We have successfully implemented the hardware configuration for gathering data sets and employing an RNN long-short-term memory (LSTM) model to determine the SoC in lithium-ion batteries, and we will discuss this in further paragraphs.

### 2.1 DAQ module

This system incorporates multiple components, such as a smart battery monitor, a controlled temperature chamber, and programmable instruments, to guarantee precise data gathering and immediate analysis. The experimental setup discussed in this work is an intelligent battery monitor equipped with sensors to precisely measure Current, Voltage, and Temperature. These sensors are interfaced with an 18 650 Li-Ion battery, enabling uninterrupted monitoring of these crucial parameters. The data obtained from the smart battery monitor is then passed through signal conditioners so that the analog-to-digital converters in the Edge devices can handle them properly. This data is essential for training the RNN LSTM model, as it supplies the required inputs for precise state-of-charge (SoC) estimation.

**Fig. 2:**
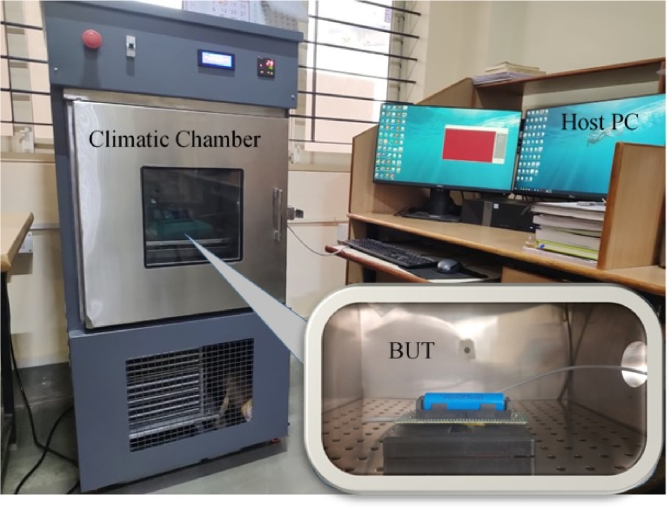
Single cell battery under test inside a temperature Chamber. Battery parameters are monitored and recorded in the Host PC.

The Battery Under Test shown in Fig. 2, which is a 18 650 Li-Ion battery is placed within a climatic chamber to replicate various environmental conditions. This chamber regulates the temperature near the battery, guaranteeing that the data obtained accurately represent various real-life situations. The climatic chamber’s controlled environment is crucial for comprehending the impact of temperature fluctuations on the battery’s performance and SoC.

### 2.2 Dataset generation procedure

The Experimental setup for the Dataset generation procedure incorporates programmable power supply and load devices. The KEITHLEY 2231A-30-3 programmable power supply delivers controlled charging cycles to the battery, while the GW-INSTEK PEL-3031E programmable load replicates different discharging scenarios. These instruments guarantee that the battery undergoes consistent and repeatable charge and discharge cycles, resulting in dependable data for model training.

**Fig. 3:**
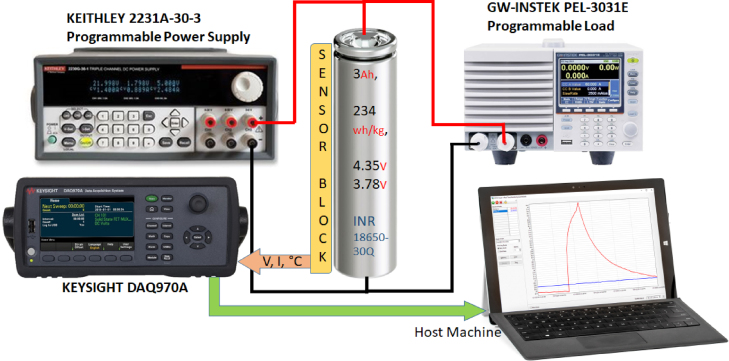
Battery parameters are monitored and recorded in the Host PC.

The KEYSIGHT DAQ970 A, a data acquisition system (DAQ), is employed for the purpose of gathering and documenting data obtained from the sensors. This system records the measurements of voltage, current, and temperature over a period of time and stores them for later analysis. The process of acquiring data is essential for constructing a resilient dataset that can be utilized to train the RNN LSTM model. The Fig. 3 shows the detailed dataset generation procedure.

These sensors are interfaced with an 18 650 Li-ion battery to carry out continuous monitoring of the said important parameters. The data from the smart battery monitor is then passed through signal conditioners so that the analog-to-digital converters in the edge devices handle them properly. This data is necessary for model training, as it provides the necessary inputs to obtain accurate state-of-charge estimates.

The battery under test is housed inside a climatic chamber to stimulate different climate conditions. Through this chamber, temperature around the battery is controlled in such a way that the acquired data are indeed a realistic variety of situations. Such variation of temperature allows an understanding of battery behavior and performance with a varying state of temperature. The setup also includes programmable power supply and load devices; that is, the KEITHLEY 2231A-30-3 programmable power supply supplies the battery with controlled charging cycles, while the GW-INSTEK PEL-3031E programmable load simulates different discharging scenarios. These tools guarantee that the battery is cycled both charge and discharge reliably and reproducibly, thus ensuring reliable data during model development. The data-acquisition system in use is the KEYSIGHT DAQ970 A, responsible for data input of the sensors and recording the same. The system integrates and stores readings for current, voltage, and temperature over time. As mentioned previously, the data acquisition methodology is crucial to creating a strong dataset that could be used in training the RNN LSTM model.

An embedded Microcontroller is hosted as the final component of the architecture, which is an edge device. The trained RNN LSTM model will be executed in the edge device which also processes the data received from the smart battery monitor in real-time. By deploying the model on the edge device, the system is able to timely provide predictions for the State of Charge (SoC) of the battery, which enables an adequate and timely action regarding battery management.

### 2.3 The current sense circuit

The current sense circuit depicted in the schematic, uses an operational amplifier (op-amp), specifically the AD8609, to measure the current flowing through a load. the key component central to the design is the sense resistor R5, which has a low resistance value of 0.1Ω but is capable of handling high wattage to accurately sense the current without significant power dissipation. This resistor is strategically placed in series with the load $R_{L}$, ensuring that all the current flowing from the battery $V_{bat}$ to the load passes through it. The voltage drop across R5 is directly proportional to the current flowing through the circuit.

To understand the circuit operation, consider that the maximum current the circuit is designed to handle is 3 A, which corresponds to the full capacity of the 18 650 lithium-ion battery (3000 mAH). At this maximum current, the voltage drop across $R_{5}$ will be V=I∗R=3A∗0.1Ω=0.3V. This voltage drop is then fed into a differential amplifier configuration using the AD8609 op-amp.

**Fig. 4:**
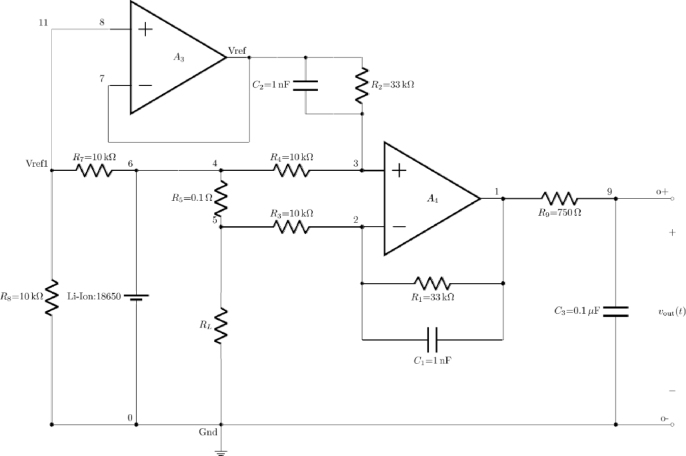
Current sense amplifier using Op-Amp AD8609.

The differential amplifier formed by the op-amp AD8609 amplifies the voltage difference between its inverting and non-inverting inputs is shown in Fig. 4. The non-inverting input (+) of the op-amp is connected to the junction between resistors $R_{3}$ and $R_{4}$, while the inverting input (−) is connected to the junction between $R_{5}$ and $R_{6}$. These resistors form a voltage divider network that scales and balances the input signals to the op-amp, ensuring that the small voltage drop across $R_{5}$ is accurately amplified.

Resistors $R_{1}$ and $R_{2}$, along with capacitors $C_{1}$ and $C_{2}$, are crucial in setting the gain and filtering the signal. The gain of the differential amplifier is determined by the ratio of these resistors. For example, if $R_{1}$ and $R_{2}$ are both 33kΩ, the gain can be calculated using the standard differential amplifier gain formula Gain=1+(R2/R1). In this case, the gain would be 2, meaning the output voltage of the op-amp would be twice the voltage difference between the inputs. The capacitors $C_{1}$ and $C_{2}$ help filter out high-frequency noise, stabilizing the circuit and ensuring a clean output signal.

The output of the differential amplifier is then passed through resistor $R_{9}$ to the final output $V_{out}$. Capacitor $C_{3}$ is placed in parallel with the output to provide additional filtering, smoothing the output signal and eliminating any residual noise. This filtered output voltage $V_{out}$ is directly proportional to the current flowing through the sense resistor $R_{5}$.

The reference voltage $V_{ref1}$, established by resistors $R_{7}$ and $R_{8}$, ensures the proper biasing of the op-amp. This reference voltage sets a baseline for the differential measurement, ensuring that the op-amp operates within its linear range and providing a stable reference point for the voltage drop across $R_{5}$.

The resistor $R_{6}$ is designed to ensure that at maximum current (3 A), the circuit remains within safe operational limits. It complements the sense resistor $R_{5}$ by limiting the current flow and providing a protective measure against overcurrent conditions. Given the battery’s capacity (3000 mAH), the circuit is designed to handle the maximum current output without exceeding the safe limits of the components.

### 2.4 The voltage sense circuit

**Fig. 5:**
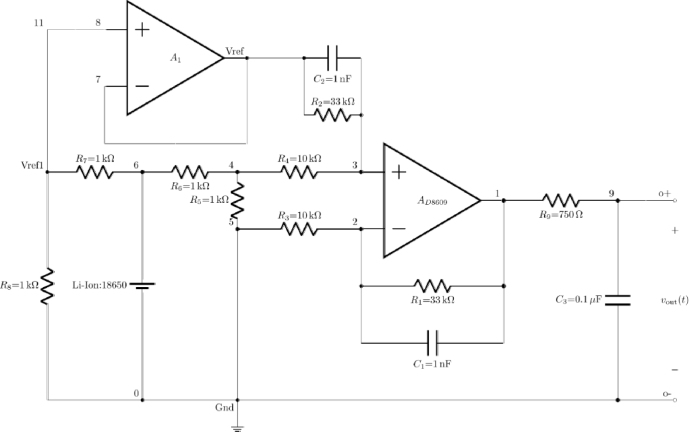
Voltage sense amplifier using Op-Amp AD8609.

The voltage sense circuit shown in the schematic is meticulously designed to measure and monitor the voltage of a power source, such as a battery, converting this measurement into a proportional output voltage suitable for various applications. At the heart of the circuit is an operational amplifier (op-amp), specifically the AD8609, configured as a differential amplifier. The schematic is shown in Fig. 5. The circuit employs a voltage divider network formed by resistors $R_{5}$ and $R_{6}$, which scale down the input voltage from the battery $V_{bat}$ to a level that the op-amp can accurately process. This is crucial as the direct battery voltage may exceed the operational range of the op-amp, necessitating this scaling down for accurate measurement. The potential divider works by dividing the input voltage in proportion to the resistance values of $R_{5}$ and $R_{6}$. For example, if $R_{5}$ and $R_{6}$ are of equal value, the voltage at the node will be half of $V_{bat}$.

The non-inverting input of the op amp (+) is connected to the midpoint of the voltage divider, ensuring that it receives a scaled version of the battery voltage. Meanwhile, the inverting input (–) of the op-amp is connected to another voltage divider formed by resistors $R_{3}$ and $R_{4}$. These resistors set up the differential amplifier’s feedback network, defining its gain. The gain of the differential amplifier can be adjusted by selecting appropriate values for these resistors, following the formula Gain=1+(R2/R1), where $R_{1}$ and $R_{2}$ are the feedback resistors in the op-amp circuit. This allows the output voltage to be a scaled and amplified version of the input voltage difference, providing a precise representation of the battery voltage.

To ensure the op-amp operates within its optimal range and provides a stable output, a reference voltage $V_{ref1}$ is established using resistors $R_{7}$ and $R_{8}$. These resistors create a stable voltage reference that biases the non-inverting input of the op-amp. This reference voltage is critical for maintaining the accuracy and stability of the differential amplifier, especially in fluctuating conditions. The reference voltage is derived from the battery voltage, with $R_{7}$ and $R_{8}$. chosen to provide a specific fraction of $V_{bat}$. This reference ensures that any variations in the battery voltage are accurately reflected in the output, without causing instability in the op-amp.

The capacitors $C_{1}$ and $C_{2}$ are strategically placed in the circuit to filter out high-frequency noise, which could otherwise distort the measurement. These capacitors smooth the input signals, ensuring that the op-amp receives a clean and stable voltage for amplification. This filtering is crucial in environments with significant electrical noise, such as those involving power electronics or RF signals. The differential amplifier output is then passed through $R_{9}$ to the final output node, with $C_{3}$ providing additional filtering to smooth the output signal further. This ensures that the output voltage$V_{out}$ is not only accurate but also stable and free from high-frequency noise.

The design also considers the practical aspects of the circuit’s operation. For instance, the selection of $R_{5}$ and $R_{6}$ ensures that the potential divider network scales the input voltage appropriately, while the feedback network of $R_{3}$ and $R_{4}$ determines the amplifier’s gain to match the desired output range. This careful selection and balancing of resistor values ensure that the circuit can handle a wide range of input voltages while providing a precise output.

## 3 Design files summary

*CAD Design Folder: Contains the 3D models and mechanical design schematics for the SCUD, created in a CAD software. These files detail the physical dimensions, structure, and assembly of the device components (see* Table 1*).*

*KiCAD Design Folder: Includes the PCB schematics and layout files for the electronic circuitry of SCUD. These files provide the designs for the hardware components and their connections.*

**Table 1:** Repository structure and access details for SCUD design files.

Design filename	File type	Open source license	Location of the file
*CAD Design Folder*	*CAD Files*	*Creative Commons Attribution 4.0 International Public License*	https://osf.io/sq8pj/files/osfstorage/6755cf83e83a75c8645636d1

*KiCAD Design Folder*	*KiCAD Design Files*	*Creative Commons Attribution 4.0 International Public License*	https://osf.io/sq8pj/files/osfstorage/6755d08214881f467f661542

*ML Model Python Files*	*Machine Learning Model Files*	*Creative Commons Attribution 4.0 International Public License*	https://osf.io/sq8pj/files/osfstorage/6755db1972df584b6003d943

*ML Model Python Files: Comprises the Python scripts used to train and deploy the LSTM-based SoC estimation model. These files include data preprocessing, model training, and the optimized model for deployment on the Arduino Nano 33 BLE.*

### 3.1 PCB design files

**Fig. 6:**
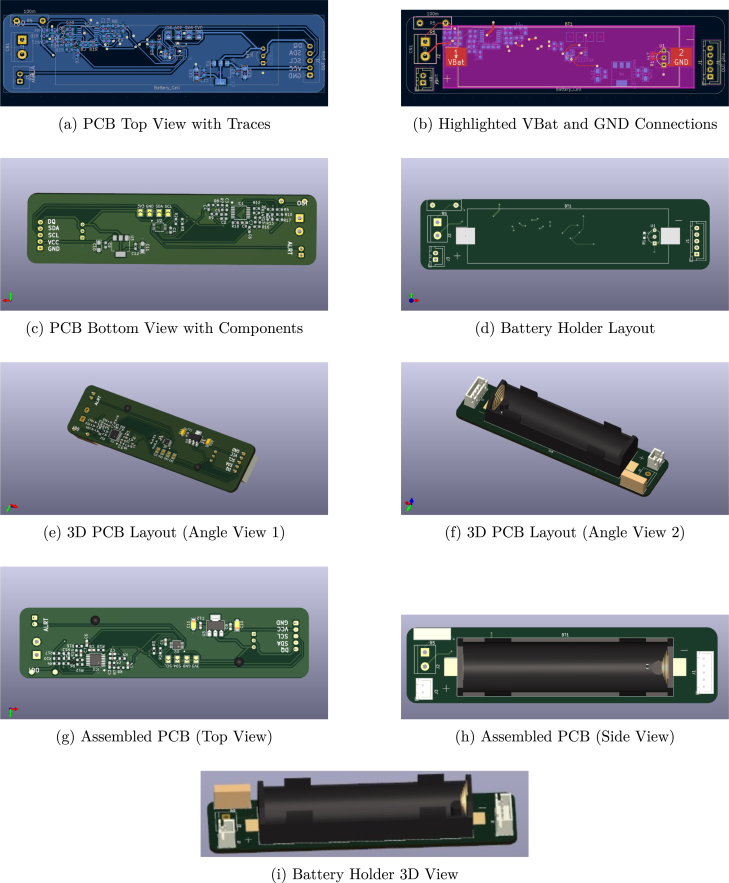
PCB Images showing different views and layouts.

The printed circuit board (PCB) is fabricated to meet specific design and quality requirements, adhering to industry standards (IPC Class 2). Below is a detailed technical description: The PCB features high-quality solder masking with excellent adhesion, ensuring durability and reliability during operation. Table 2 gives a detailed approach to PCB manufacturing protocol. The use of FR4 material with a glass transition temperature of 130°C makes the PCB suitable for moderate thermal environments, typical for battery monitoring applications. The boards underwent rigorous QA testing to ensure they meet the required dimensional and material specifications. This high-quality PCB is optimized for use in the Battery Monitor project, providing robust electrical performance and durability. Its precise dimensions and adherence to IPC Class 2 standards ensure reliable operation in demanding applications. Fig. 6 shows the design files generated from KiCAD software:(a) PCB Top-View with Traces, (b) Highlighted $V_{Bat}$ and GND Connections, (c) PCB Bottom View with Components, (d) Battery Holder Layout, (e) 3D PCB Layout (Angle View 1) (f) 3D PCB Layout (Angle View 2), (g) Assembled PCB (Bottom View), (h) Assembled PCB (Top View), and (i) Battery Holder 3D View.

**Table 2:** PCB specifications.

PCB specifications	
General specifications	Dimensional specifications

Customer name: MSRIT	Length: 27.1 mm ± 0.15 mm
Project name: Battery monitor	Width: 111.05 mm ± 0.15 mm
PCB type: 2 layers	Thickness: 1.6 mm ± 10%
PCB size: 30.09 cm${}^{2}$	**Surface finish**
Service type: Standard service	HASL (Hot Air Solder Leveling)

Solder mask specifications	Legend/Print specifications

Adhesion test: Yes	Print color: White
Mask color: Green	Print sides: Both sides

Electrical test results	Material properties

Units tested: 5	Laminate material: FR4
Units passed: 5	Laminate Tg: Typ 130°C
Units rejected: 0	Copper thickness: 35 μm (1 oz)

### 3.2 Schematics

The schematic diagram of the SCUD (Smart Culinary Utility Device) is shown in Fig. 8. It shows how the signal converter was built using the AD8609 operational amplifier, the ADC (ADS1115), and an OLED display, all of which were connected to the Arduino Nano 33 BLE edge device.

### 3.3 CAD designs

**Fig. 7:**
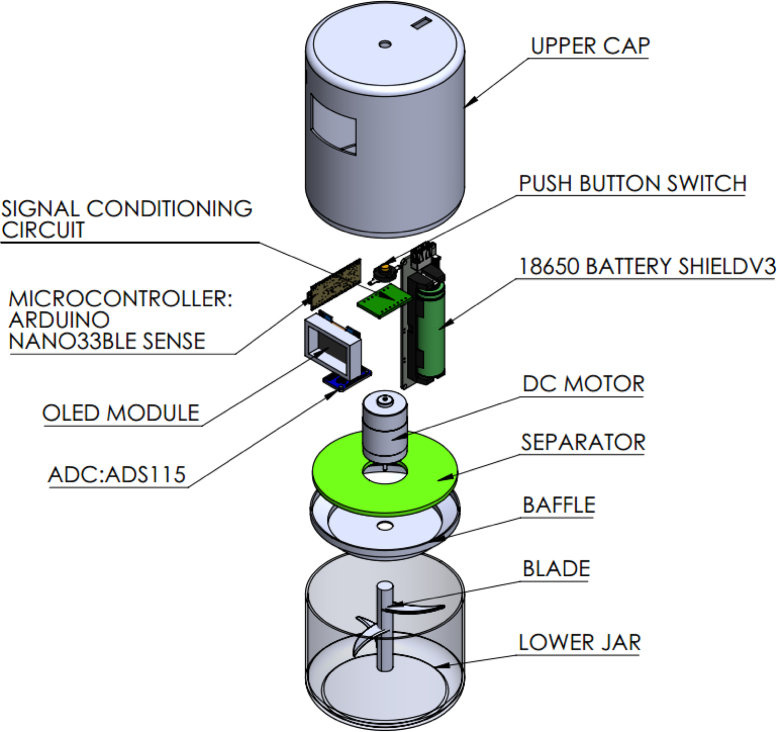
SCUD: Smart Culinary Utility Device, exploded view.

The CAD design is shown in Fig. 9. Items (a, b, c) represent the Front View (FV), Side View (SV), and Top View (TV) of the Upper Cap, respectively. Item (d) shows the Top View (TV) and Side View (SV) of the Separator. Subfigures (e and f) illustrate the Side View (SV) and Top View (TV) of the Baffle. Subfigures (g and h) present the Side View (SV) and Top View (TV) of the Lower Jar. Finally, subfigures (i, j, k, and l) depict the isometric views of the upper cap, lower jar, baffle, and separator, respectively. Finally, Fig. 7 shows the Exploded View of the SCUD: Smart Culinary Utility Device.

## 4 Bill of materials

A detailed Bill of materials is shown in Table 3. the components are mainy procured from the local Indian market. the cost of the total project is projected in ₹(INR: Indian National Rurees).

## 5 Build instructions

The SCUD hardware design is a compact PCB-based solution integrating battery monitoring, power management, and communication capabilities. Below are detailed step-by-step instructions for reproducing the design, referencing the shared design files and images.

### 5.1 Materials and components

Before starting the build, gather all the required components. These include a custom-designed PCB, as detailed in the KiCAD files and 3D models, and electronic components such as an Arduino Nano 33 BLE microcontroller module, AD8609 Op-Amp module, 100 mΩ shunt resistor-type current sensors, a 18 650-compatible battery holder, a 4.7 kΩ NTC temperature sensor, an SSD1306 OLED module, and an ADS1115 analog-to-digital converter. Additional components include power and alarm LEDs, JST connectors, pin headers, terminal blocks, and other resistors, capacitors, and ICs specified in the schematic. Essential tools include a soldering station, multimeter, screwdriver set, protective eyewear, gloves, and heat shrink tubing.

**Table 3:** BOM: Bill of Materials in ‘₹’ INR: Indian National Rupee.

S.No.	Designator	Value	Component	Quantity	Cost/Unit (₹)	Total cost (₹)	Source of materials	Material type
1	IC	AD8609	SMD, OPAMP, QUAD, 12uV Vos, TSSOP-14	1	416.00	416.00	https://in.element14.com	Semiconductor
2	RES	10KΩ	SMD, 10KΩ, RES, 0603, 0.1%, 0.1W	2	13.36	26.72	https://in.element14.com	Thin-film (Sulfur)
3	RES	1KΩ	SMD, 1KΩ, RES, 0603, 0.1%, 0.1W	7	29.22	204.54	https://in.element14.com	Thin-film
4	RES	33KΩ	SMD, 33KΩ, RES, 0603, 0.1%, 0.1W	2	13.36	26.72	https://in.element14.com	Thin-film
5	RES	750R	SMD, 750Ω, RES, 0603, 0.1%, 0.1W	2	14.20	28.40	https://in.element14.com	Thin-film
6	RES	100KΩ	SMD, 100KΩ, RES, 0603, 0.1%, 0.1W	2	13.30	26.60	https://in.element14.com	Thin-film
7	RES	75KΩ	SMD, 75KΩ, RES, 0603, 0.1%, 0.1W	2	7.46	14.92	https://in.element14.com	Thin-film
8	IC	DS18B20	Temperature sensor, ±0.5°C, −55 to +125°C	1	119.00	119.00	https://robu.in	Semiconductor
9	RES	1.2KΩ	SMD, 1.2KΩ, RES, 0603, 1%, 0.1W	1	11.70	11.70	https://in.element14.com	Thin-film
10	RES	100R	SMD, 100Ω, RES, 0603, 1%, 0.1W	1	13.30	13.30	https://in.element14.com	Thin-film
11	CAP	0.1μF	SMD, 0.1μF, 0603, 10%, 16 V, X7R	2	2.20	4.40	https://in.element14.com	Ceramic type
12	CAP	10μF	SMD, 10μF, 0805, 10%, 16 V, X7R	1	7.30	7.30	https://in.element14.com	Ceramic type
13	IC	TP4056	Battery charging module, Linear method, 4.2 V, 4.2W	1	35.00	35.00	https://robu.in	Semiconductor
14	Battery	18 650	Li-Ion Battery, 3.6 V, 3000mAh	1	949.00	949.00	https://robu.in	Li-Ion
15	Sense	0.1Ω	SMD, CURRENT SENSE RES, 100mΩ, 2512, 1%, 3W	1	24.89	24.89	https://in.element14.com	Metal strip

**Total amount in INR:**						₹1908.50

**Fig. 8:**
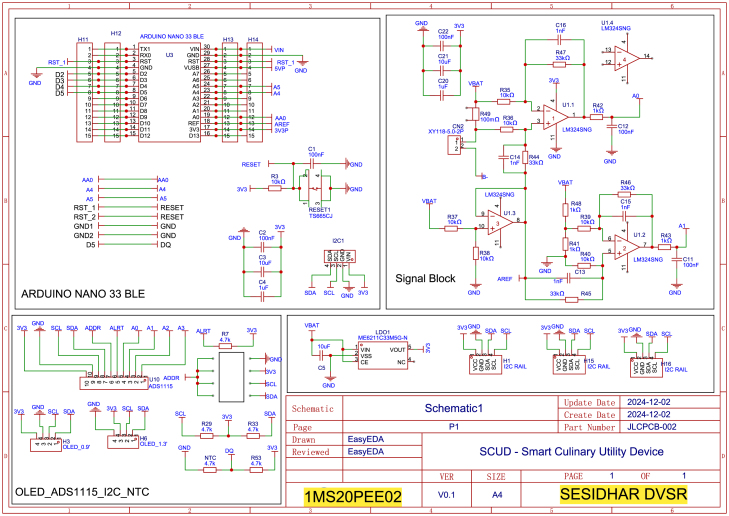
SCUD: Smart Culinary Utility Device schematic diagram.

**Fig. 9:**
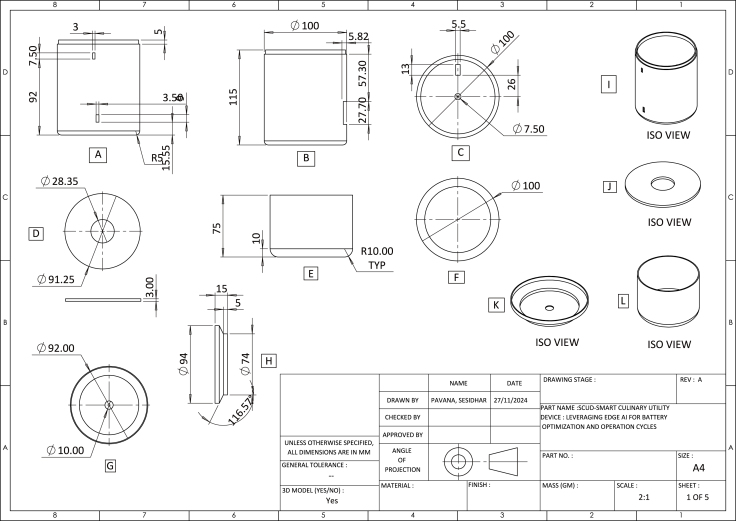
SCUD: Smart Culinary Utility Device CAD designs.

**Electronic Components:**

• Arduino Nano 33 BLE Microcontroller module
• Op-Amp module AD8609
• Shunt Resistor type Current Sensors (100 mΩ)
• Battery Holder (18 650 Li-ion compatible)
• NTC Temperature sensor (4.7 kΩ)
• OLED Module (SSD1306)
• ADS1115, Analog to Digital Converter
• AMS1117-3.3 voltage regulator
• LEDs (Power and Alarm)
• Connectors: JST connectors (×2), pin headers, and terminal blocks.
• Resistors, capacitors, and ICs as specified in the schematic.

### 5.2 PCB assembly

Begin by inspecting the PCB for defects such as shorts or breaks in the traces. Use the top and bottom view images (Fig. 6, Fig. 6) to identify the placement of components. Populate the PCB with smaller components first, such as resistors and capacitors, soldering them in place according to the KiCAD schematic. Next, mount the ICs and sensors, including the AD8609 Op-Amp, ADS1115 ADC, and AMS1117-3.3 voltage regulator, ensuring proper orientation to prevent damage. Attach the JST connectors for the battery holder and output connections, and solder pin headers for microcontroller module interfaces. Install the LEDs, connecting the green LED for power and the red LED for alarms, with the cathode (short leg) linked to the ground plane. Secure the battery holder using screws and nuts, aligning it as per the 3D model illustrations (Fig. 6, Fig. 6). Solder all components using a fine-tipped soldering iron, checking for cold solder joints or bridges between traces. Finally, inspect all connections using a multimeter to confirm there are no shorts and verify alignment with the schematic.

### 5.3 System integration

After assembling the PCB, plug in the microcontroller module, OLED display, and NTC sensor into their respective headers. Use JST connectors to attach the battery holder. Connect the output pins (DQ, SDA, SCL, VCC, GND) to external systems or test loads, referring to Fig. 6(f) for proper labeling. Insert the 18 650 battery into the holder and verify the output voltage using a multimeter. Test the functionality of the AD8609 and ADS1115 signal conditioner for current and voltage readings, confirm timestamp functionality, and ensure the NTC sensor outputs accurate temperature data.

### 5.4 Design decisions

Key design decisions include selecting a battery holder compatible with 18 650 Li-ion batteries for extended power supply, using a power converter to maintain a steady 3.3 V output despite battery discharge levels, and optimizing the PCB layout for minimal noise and efficient routing. Clear silkscreen markings on the PCB further enhance assembly accuracy.

### 5.5 Safety concerns

To ensure safety, always use protected 18 650 batteries to prevent overcharging or overheating. Avoid short circuits by insulating battery holder connections. Conduct soldering in a well-ventilated area, wearing protective gloves and eyewear to prevent inhaling fumes or exposure to soldering hazards. During electrical testing, use low-current settings on the power supply to minimize the risk of component damage, and double-check polarity before powering the system.

### 5.6 References to design files

Refer to the shared KiCAD layout (6(a), 6(c)) for PCB trace details and Figs. 6(d), 6(e), 6(i) for insights into component placement and physical assembly. The KiCAD schematic provides detailed component connections for further clarity.

### 5.7 Final assembly

Once all components are assembled, enclose the system in a protective casing, ensuring adequate ventilation for heat dissipation. Secure the PCB and battery holder in place using M3 screws. Perform a final system check to verify that all modules operate as intended. By following these detailed instructions, the SCUD hardware design can be faithfully reproduced, ensuring functionality and safety.

By following these instructions, the hardware design can be faithfully reproduced while ensuring functionality and safety.

## 6 Operation instructions

The SCUD hardware system is designed to operate safely and efficiently, with clear guidelines for setup, operation, and maintenance. These instructions ensure optimal use while addressing potential safety hazards.

### 6.1 Initial setup

Begin by placing the SCUD hardware on a non-conductive, flat surface to prevent accidental short circuits. Ensure all components, such as the battery holder, OLED display, and connectors, are securely mounted. Insert a fully charged 18 650 Li-ion battery into the holder, ensuring the correct polarity alignment: the positive terminal of the battery should match the “+” symbol on the holder. Incorrect polarity can cause circuit damage or overheating. Connect external interfaces, including sensors or additional modules, to the appropriate output terminals. The pin mapping for connections is as follows: DQ for data output (NTC sensor), SDA for serial data in I2C communication, SCL for the I2C serial clock, VCC for power supply, and GND for ground. Use insulated wires for these connections to prevent exposed wiring hazards.

• **Pin Mapping:**
  – DQ: Data output (NTC sensor)
  – SDA: Serial data for I2C communication
  – SCL: Serial clock for I2C
  – VCC: Power supply
  – GND: Ground
• **Safety Note:** Use insulated wires to avoid exposed connections.

### 6.2 Powering up

To power the system, press the “Push to ON” power switch. Verify the green Power LED to confirm successful operation. If the LED does not light up, check the battery connections and polarity. Upon successful initialization, the OLED display will present battery parameters, including voltage (in volts), current consumption (in milliamps), temperature (in degrees Celsius), and the State of Charge (in percentage). If the OLED display does not activate, inspect the module connections and confirm the functionality of the microcontroller.

### 6.3 Functional operations

The SCUD system continuously monitors and displays the battery’s voltage and current using the ADS1115 ADC. Voltage readings should remain within the range of 3.0–4.2 V for standard Li-ion batteries, while current measurements depend on the connected load. Disconnect the load immediately if the current exceeds the rated capacity of the shunt resistor to avoid damage. Temperature is monitored via the NTC sensor, with readings displayed on the OLED. If the temperature exceeds 60°C, disconnect the power source and investigate overheating components. Additionally, the system enables real-time data logging of voltage, current, and temperature, saving this information in a .csv file with a timestamp on the host machine. Ensure files are saved properly after system shutdown to prevent data corruption. The red Alarm LED activates to indicate faults such as battery overcurrent, overheating, or low voltage (<3.0V). In such cases, immediately turn off the system and resolve the issue before resuming operations.

### 6.4 Turning off the system

To power down, release the “Push to ON” switch, ensuring the Power LED turns off. If the system will not be used for an extended period, remove the battery and store it in a cool, dry place. Disconnect all external modules or wires, securing loose wires to prevent damage or accidental short circuits.

### 6.5 Maintenance and safety tips

Regular maintenance includes cleaning the PCB and components with a dry, soft brush to remove dust, inspecting solder joints for cracks or corrosion, and verifying the security of all connectors and headers. For battery handling, avoid using damaged or swollen batteries and never short-circuit the terminals. Overcurrent protection involves avoiding loads exceeding the system’s capacity, and overheating can be managed by monitoring sensor readings and operating in well-ventilated areas. During repairs, ensure soldering is conducted in a well-ventilated space to mitigate fume exposure.

### 6.6 Troubleshooting guide

Table 4 shows the troubleshooting guide.

**Table 4:** Troubleshooting guide for SCUD hardware.

Issue	Possible cause	Solution
Power LED does not light up	Dead or incorrectly inserted battery	Replace or reinsert the battery correctly.
OLED display is blank	Faulty connection or loose header	Check and reseat the OLED header.
Alarm LED stays on	Overcurrent, overheating, or low voltage	Disconnect power and check the system.
No voltage/current readings	ADS1115 not soldered properly	Inspect and re-solder the ADC module.

**Fig. 10:**
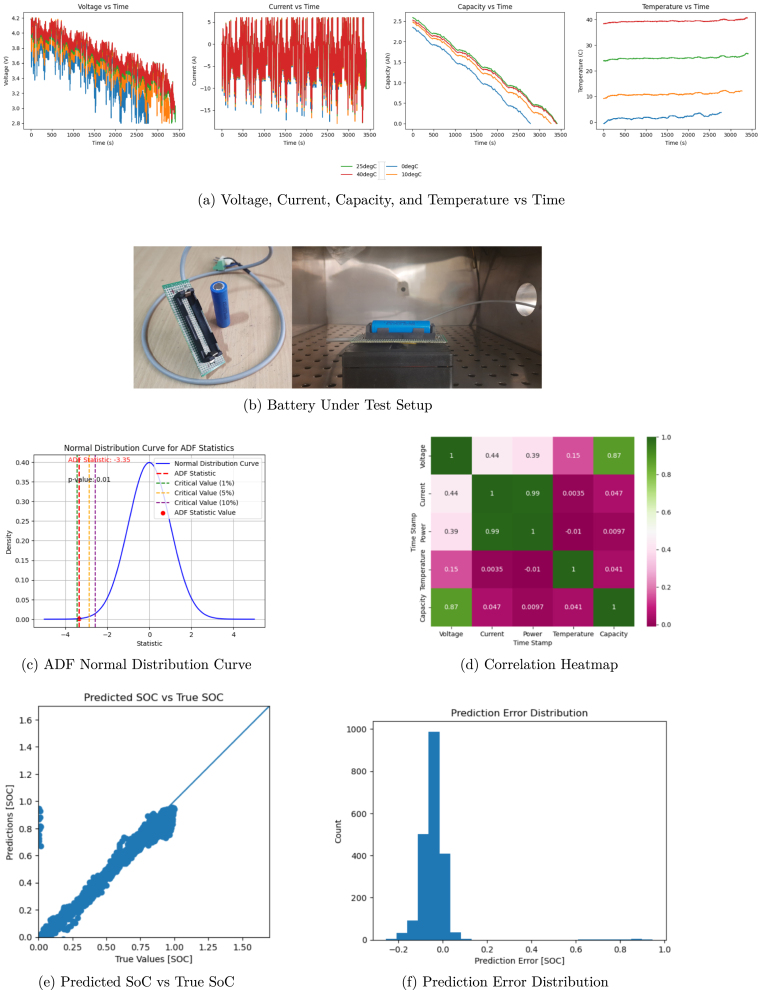
Visualization of experimental setup, analysis, and model results.

**Fig. 11:**
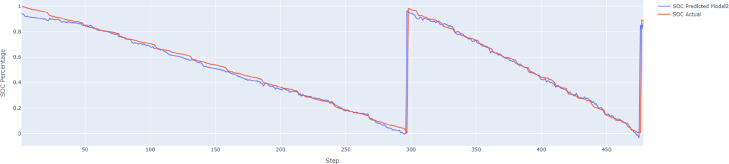
Predicted SoC vs. Actual SoC.

## 7 Validation and characterization

The Fig. 10 collectively illustrate the experimental setup, analysis, and results for a battery’s State of Charge (SoC) estimation model.

The first set of images provides a comprehensive view of the data and model analysis:

• The **Voltage, Current, Capacity, and Temperature vs. Time** plots demonstrate the battery’s behavior during charge–discharge cycles. These plots highlight key dynamics, including voltage drop during discharge, fluctuating current under varying loads, the linear decrease in capacity, and stable temperature profiles. These trends are critical for validating the model’s alignment with physical battery behavior. Fig. 10(a) shows the battery parameters.
• The **Battery Under Test Setup** displays the hardware arrangement, including the battery holder and the controlled environment for data collection. This setup ensures accurate and reliable acquisition of data. Fig. 10(b) shows the battery under test.
• The **ADF Normal Distribution Curve** verifies the stationarity of the time-series data, with a p-value and critical thresholds confirming the dataset’s suitability for predictive modeling. Fig. 10(c) shows the ADF Test curve.
• The **Correlation Heatmap** identifies relationships between parameters such as voltage, current, capacity, and temperature. Strong correlations, such as between voltage and capacity, inform the selection of significant features for the SoC prediction model. Fig. 10(d) shows the correlation matrix of data set features.
• The **Predicted SoC vs. True SoC Scatter Plot** demonstrates the model’s accuracy, with data points closely aligned along the diagonal, indicating strong agreement between predictions and actual values. Fig. 10(e) shows the predicted SoC vs. True SoC distribution.
• The **Prediction Error Distribution** plot shows a narrow error range centered around zero, confirming the precision and reliability of the SoC estimation model. Fig. 10(f) shows the prediction error distribution.

Fig. 11 presents a line plot comparing the **Predicted SoC** with the **Actual SoC** over time. The red and blue lines closely overlap, showcasing the model’s high accuracy in estimating SoC during dynamic battery operations. The clear alignment between predicted and actual values highlights the robustness of the model across varying conditions.

## 8 Conclusion and future work

**Conclusion**

This paper presents a robust framework for estimating the State of Charge (SoC) of a Li-ion battery using machine learning techniques and an experimental hardware setup. By integrating real-time data collection with advanced predictive modeling, the framework demonstrates high accuracy in SoC estimation under varying operational conditions. The experimental results, validated through statistical tests and error distribution analysis, confirm the effectiveness of the proposed model. The close alignment between predicted and actual SoC values underscores the model’s reliability and precision.

The correlation analysis highlights the significant relationships between voltage, current, capacity, and temperature, guiding feature selection for the predictive model. The model’s performance is further validated through error distribution and time-series plots, which exhibit minimal prediction errors and strong consistency with the battery’s physical behavior during charge–discharge cycles. The designed hardware setup, coupled with an efficient data acquisition system, ensures the quality and reliability of the collected data, providing a strong foundation for predictive modeling.

This work contributes to the field of battery management systems (BMS) by offering a scalable, data-driven approach for SoC estimation, which is essential for enhancing the safety, efficiency, and longevity of batteries in various applications, including electric vehicles and renewable energy storage.

**Future Work**

Building on the findings of this research, several directions can be pursued to enhance the proposed framework:

1. **Incorporation of More Diverse Datasets:** Expanding the dataset to include a wider range of operational conditions, such as varying temperatures, discharge rates, and battery chemistries, to improve the model’s generalizability.
2. **Integration with Digital Twin Technology:** Developing a digital twin of the battery system to simulate and test the performance of the SoC model under different scenarios, enabling proactive decision-making and system optimization.
3. **Exploration of Advanced Algorithms:** Investigating advanced machine learning and deep learning models, such as ensemble techniques or recurrent neural networks, to further enhance the accuracy and robustness of SoC prediction.
4. **Inclusion of Aging Effects:** Extending the model to account for battery aging and degradation over time, enabling predictive maintenance and better lifecycle management.
5. **Real-Time Implementation:** Developing a complete real-time SoC monitoring and control system integrated with battery management systems for real-world applications in electric vehicles and energy storage systems.

By addressing these future directions, the framework can be further refined to meet the growing demands of advanced battery management systems, ensuring reliability and scalability in dynamic environments.

## CRediT authorship contribution statement

**Sesidhar DVSR:** Writing – review & editing, Writing – original draft, Visualization, Software, Project administration, Methodology, Investigation, Conceptualization. **Chandrashekhar Badachi:** Writing – review & editing, Supervision, Resources, Project administration, Formal analysis, Data curation, Conceptualization. **Chandrashekar Nagawaram:** Visualization, Validation, Supervision, Software, Project administration, Methodology, Formal analysis, Conceptualization. **Panduranga Chary Kondoju:** Validation, Project administration, Methodology, Investigation, Funding acquisition, Conceptualization. **Pavana Salunkhe:** Validation, Project administration, Methodology, Conceptualization. **Sahil Kumar Chaurasia:** Validation, Software, Methodology.

## Ethics statements

The authors declare that no human beings or animals were used in this work.

## Funding

This research work is supported by the Ramaiah Institute of Technology , Affiliated to Visvesveraya Technological University, Belagavi. Bharat (India). 590018.

## Declaration of competing interest

The authors declare no competing interests to the best of their knowledge.
